# Evolution in Dentistry

**Published:** 1890-11

**Authors:** J. A. Robinson


### Evolution in Dentistry.

BY J. A. ROBINSON, D.D.S.

Evolution is the finding of a new phaze of an old truth, thoughts are the blossoms and our acts are the fruitage of the truths that go forth into uses. In this way we make progress in mechanics and the arts and in science. As in plant life we do not always see the whole process, and as in animal life every new birth seems almost a miracle; and it is as sudden when a completed thing as when the butterfly comes out of the chrysalis or when the water turns to steam by the raising of the temperature, which is a gradual process and corresponds to the growth of the arts and sciences of which dentistry forms a part. It is the result of the civilization in which we live. When old Dr. Tilden came to Cleveland in 1856 or 1857, he had no idea of the vast amount of oxyphosphate cement that would be used in dentistry in the short period of thirty years. It was not introduced as a capping for exposed pulps at first, but as a new and permanent filling for the teeth. When by experiment it was found that in conjunction with carbolic acid it was painless and the pulp remained alive, (though it has been doubted by good operators) its use became somewhat general and it is now one of the most potent factors in relief of pain in the general process of saving teeth.

This process is perfectly natural and is in accord with the general law of evolution which teaches that improvements always grow out of the conditions immediately preceeding. This is largely true in regard to the porcelain art process as practiced by Dr. C. H. Land, of Detroit. Whether Dr. Land was the original inventor of the platinum matrix or not, I do not know. I have heard it questioned. Of this it is certain that he obtained a patent for porcelain inlays and has the credit of the invention and process. The porcelain inlay was only a half step in the improvement that has been attained.

Dr. Albert Robinson, of Grand Rapids, after buying the Land process and finding that it was too difficult for ordinary operators,

(as the furnace could not, without some stronger power than the foot bellows to fuse the porcelain, be worked in the ordinary dental office) conceived the idea of filling the matrix with gold or some other metal and thus make a filling that could be made in any ordinary dental office, and it had another advantage of an undercut round the border of the filling and cuts across the under side made with a saw such as used by jewelers, or a thin file whereby the plug would be very much strengthened at the point of connection with the cavity, and entirely shut out the secretions of the mouth which are liable to wash out the cement that holds the filling in place.

Dr. Robinson has a patent for the metallic filling and a combination of metals that can be fused with an ordinary alcohol lamp in any dental office that he calls the Robinson method of filling teeth.

After I bought the patent process of Dr. Land and Dr. Pretty (who was Land's assistant in his office) came to Jackson to assist me in working the Land furnace, I showed him the process of filling the matrix with gold as an easier way and just as good as the porcelain; he told me that he had never seen or thought of it and that the Land plan was to do away with gold fillings altogether and drive the jewelry work out of the mouth and Dr. Land so advertised it in his circulars. I have made some porcelain work that was very satisfactory, but it corresponds to continuous gum work for sets of teeth it will not do for the million because it is altogether out of their reach.

The entire community need the services of a good dentist from the six year old child to the aged person, and the dentist and the method that can prevent decays and alleviate pain and save the teeth will be the one that is fittest to survive and will live.

It is now twenty years since I published in a dental journal that carbolic acid paralyzed the dental pulp, but would not destroy it without repeated and continuous applications. Within the past few months I have made some further experiments that confirm what I said at that time. After removing the debris from an aching tooth, (if you find blood so much the better) apply strong carbolic acid on cotton and let it remain twenty minutes; now with a sharp burr in the engine running very fast, cut out the pulp cavity clean, then apply again carbolic acid and wait twenty minutes longer and you can remove the pulp from the canals. Take a fine barbed Donaldson nerve needle and wet it with the acid when you are ready and say to your patient something like this to prepare their mind: "Now, if you knew that you would die in one minute if you did not hold absolutely still could you do it?" The answer will always be, "I could." "Now do so and you will not feel a particle of pain after the first touch of the instrument to the nerve." This is the theory and practice. The nerve fibril always contracts on the slightest touch of any heterogenous body and gives the alarm to the transverse nerve to which it is attached and that nerve is alive and not paralyzed; now push the broach with a steady and firm hand very slowly until the needle reaches the apex of the root, and the needle forms an ecraseur and crushes it to separation; you can now wind the needle with a few fibres of cotton wet with carbolic acid and remove the whole from the canal. If you force air through the foramen it will cause pain by pressure on the transverse section but not otherwise. The fibres of a muscle or the whole body coming suddenly in contact with a cold substance receives a shock and the nerve of a tooth seems to be the most sensative part of the human organism. We do not feel or realize a law till it is broken.

Since the general acceptance of the germ theory of disease, the antidotal and antiseptic practice follows as a necessity and dentistry is carried forward by the same wave of civilization. In the enthusiasm of any new method like the Robinson method we do not wish to be understood as saying that it is absolutely the best in every case, or the only way to save teeth, but we do claim that it is an evolution in saving teeth both in time and expense. What is best for the patient must always be best for the operator and will prevent that wholesale extraction of the teeth that have been unnecessarily sacrificed in almost every dental office in the land.

The matrix is so easily adjusted to the cavity and filled and the labor is comparatively nothing when compared with filling teeth with gold or tin. There is never any inflammation of the periosteum by the protracted use of the mallet in large cavities; the average cavity can be filled in one fourth of the time occupied in the old way and from experience of more than two years they may be said to give universal satisfaction. So far I have not lost a single case. I have had to replace three per cent or three in every hundred by the breaking of the cement and that was done in a very few minutes, but have had to make only one new filling that was lost in more than two hundred fillings. Of course the matrix must be made to fit the cavity and the cement thoroughly mixed to obtain good results. I have thought Justi's cement was the strongest for general use.

Now taking into consideration the fact that the work done with the new method has invariably been done on the frailest teeth and those most liable to be troublesome, a great number on bicuspids and molars on posterior surfaces and difficult cavities to fill, I think it is a remarkable showing.

Another thing is quite certain that the teeth do not change color. When I began to fill in this way, I filled with silver coin, but that can not be successfully done as the silver destroys the platinum matrix unless it is imbeded in plaster which takes too long, but the new combination of metals if made rich enough with gold melts very easily and will not change color in the mouth.

---

Local Anæsthesia by Means of Carbonic Acid.—According to Voituriez, the anæsthetic effects of carbonic acid, described by Brown-Sequard, can be obtained in an extremely simple manner by means of the ordinary siphons containing mineral water charged with the gas. The anæsthesia is obtained by projecting at a distance the contents of two or three siphons of seltzer water, limiting the application to the part to be operated upon. The insensibility to pain lasts about five minutes and then slowly disappears. The method is chiefly applicable to the limbs, as about the head and trunk the irrigation is somewhat inconvenient.—*London Medical Record.*

